# Bumetanide Effects on Resting-State EEG in Tuberous Sclerosis Complex in Relation to Clinical Outcome: An Open-Label Study

**DOI:** 10.3389/fnins.2022.879451

**Published:** 2022-05-12

**Authors:** Erika L. Juarez-Martinez, Dorinde M. van Andel, Jan J. Sprengers, Arthur-Ervin Avramiea, Bob Oranje, Floortje E. Scheepers, Floor E. Jansen, Huibert D. Mansvelder, Klaus Linkenkaer-Hansen, Hilgo Bruining

**Affiliations:** ^1^Department of Integrative Neurophysiology, Center for Neurogenomics and Cognitive Research (CNCR), Amsterdam Neuroscience, VU University Amsterdam, Amsterdam, Netherlands; ^2^Child and Adolescent Psychiatry and Psychosocial Care, Emma Children’s Hospital, Amsterdam UMC, Vrije Universiteit Amsterdam, Amsterdam, Netherlands; ^3^Department of Psychiatry, UMC Utrecht Brain Center, University Medical Centre Utrecht, Utrecht, Netherlands; ^4^Department of Pediatric Neurology, UMC Utrecht Brain Center, University Medical Center Utrecht, Utrecht, Netherlands; ^5^N=You Neurodevelopmental Precision Center, Amsterdam Neuroscience, Amsterdam Reproduction and Development, Amsterdam UMC, Amsterdam, Netherlands; ^6^Levvel, Academic Center for Child and Adolescent Psychiatry, Amsterdam, Netherlands

**Keywords:** tuberous sclerosis complex (TSC), bumetanide, EEG, excitation-inhibition balance, repetitive behavior, irritability, open-label study

## Abstract

Neuronal excitation-inhibition (E/I) imbalances are considered an important pathophysiological mechanism in neurodevelopmental disorders. Preclinical studies on tuberous sclerosis complex (TSC), suggest that altered chloride homeostasis may impair GABAergic inhibition and thereby E/I-balance regulation. Correction of chloride homeostasis may thus constitute a treatment target to alleviate behavioral symptoms. Recently, we showed that bumetanide—a chloride-regulating agent—improved behavioral symptoms in the open-label study Bumetanide to Ameliorate Tuberous Sclerosis Complex Hyperexcitable Behaviors trial (BATSCH trial; Eudra-CT: 2016-002408-13). Here, we present resting-state EEG as secondary analysis of BATSCH to investigate associations between EEG measures sensitive to network-level changes in E/I balance and clinical response to bumetanide. EEGs of 10 participants with TSC (aged 8–21 years) were available. Spectral power, long-range temporal correlations (LRTC), and functional E/I ratio (*f*E/I) in the alpha-frequency band were compared before and after 91 days of treatment. Pre-treatment measures were compared against 29 typically developing children (TDC). EEG measures were correlated with the Aberrant Behavioral Checklist-Irritability subscale (ABC-I), the Social Responsiveness Scale-2 (SRS-2), and the Repetitive Behavior Scale-Revised (RBS-R). At baseline, TSC showed lower alpha-band absolute power and *f*E/I than TDC. Absolute power increased through bumetanide treatment, which showed a moderate, albeit non-significant, correlation with improvement in RBS-R. Interestingly, correlations between baseline EEG measures and clinical outcomes suggest that most responsiveness might be expected in children with network characteristics around the E/I balance point. In sum, E/I imbalances pointing toward an inhibition-dominated network are present in TSC. We established neurophysiological effects of bumetanide although with an inconclusive relationship with clinical improvement. Nonetheless, our results further indicate that baseline network characteristics might influence treatment response. These findings highlight the possible utility of E/I-sensitive EEG measures to accompany new treatment interventions for TSC.

**Clinical Trial Registration:**

EU Clinical Trial Register, EudraCT 2016-002408-13 (www.clinicaltrialsregister.eu/ctr-search/trial/2016-002408-13/NL). Registered 25 July 2016.

## Introduction

Tuberous sclerosis complex (TSC) is a monogenetic disorder characterized by a loss-of-function mutation in the mTOR pathway regulators TSC1 or TSC2 (tumor suppressor) genes. This mutation leads to multiorgan lesions, including brain tubers, and neuropsychiatric disorders (e.g., intellectual disability, ASD-related behavior and epilepsy) (Prather and de Vries, 2004; Curatolo et al., 2008; de Vries et al., 2015). With the recent development of mTOR inhibitors, a rational approach to alleviate both behavioral and epilepsy-related symptomatology became feasible (de Vries, 2010; Sahin, 2012; Krueger et al., 2013; Jülich and Sahin, 2014; Curatolo, 2015; Leclezio and de Vries, 2015). Yet, mTOR inhibitors have not consistently improved TSC phenotypes and the unknown long-term effects on normal development may limit their application (Sahin, 2012; Krueger et al., 2017; Overwater et al., 2019). Other targets to improve the behavioral symptoms in TSC are being considered, specifically, modulation of neuronal excitation-inhibition (E/I) balance.

Preclinical and clinical findings have implicated neuronal E/I imbalances in TSC pointing toward a reduced inhibition (White et al., 2001; Wang et al., 2007; Taki et al., 2009; Fu et al., 2012; Mori et al., 2012; Talos et al., 2012; Bateup et al., 2013; Aronica and Crino, 2014; Ruffolo et al., 2016; Nadadhur et al., 2019; Afshar Saber and Sahin, 2020; Haji et al., 2020). This may be important as E/I balance is a key principle for neuronal network organization and synchronization of neuronal oscillations, which are crucial for cognitive processes (e.g., cognitive integration, information processing, sensory binding, association, and memory) (Turrigiano and Nelson, 2004; Kinouchi and Copelli, 2006; Avramiea et al., 2020). In TSC knock-out (KO) mouse models, selective deletion of TSC1 in hippocampal pyramidal neurons resulted in deficits in the inhibitory synaptic function and an enhanced E/I ratio leading to hippocampal network hyperexcitability (Bateup et al., 2013). Other animal models with selective TSC1 gene deletion in interneuron progenitor cells resulted in reduced number of GABAergic interneurons in neocortical regions and hippocampus, altered interneuron migration and decreased seizure threshold (Fu et al., 2012). Similarly, TSC1 KO in medial ganglionic eminence (MGE)-derived (hippocampal) interneurons showed a reduction in synaptic inhibition of pyramidal cells by interneurons and associations with altered behavior in mice (Haji et al., 2020). In human tissue from the temporal lobe of a TSC patient, altered excitatory synaptic currents suggestive of ictal discharges favoring neocortical hyperexcitability and seizure generation have been reported (Wang et al., 2007). Interestingly, human-induced pluripotent stem cells (hiPSCs) from TSC patients have shown increased neuronal activity, measured by calcium transients, and higher spontaneous neuronal firing (Nadadhur et al., 2019; Afshar Saber and Sahin, 2020). Cortical tubers are a hallmark in TSC linked to epilepsy and neurobehavioral symptoms (Aronica and Crino, 2014). In humans, non-invasive measurements of GABA and glutamate levels in cortical tubers using MR spectroscopy revealed an excess of GABA molecules but decreased number of GABA_*A*_ receptors (Taki et al., 2009; Mori et al., 2012). Pathological examination of cortical tuber tissue (postsurgical excision and postmortem) has shown decreased expression of GABA_*A*_ receptor subunits (White et al., 2001; Talos et al., 2012). Importantly, tuberal tissue has shown overexpression of the chloride cotransporter NKCC1 and decreased KCC2 expression, leading to unwanted high neuronal chloride levels, and an aberrant depolarizing (excitatory) effect of GABAergic signaling (Talos et al., 2012; Ruffolo et al., 2016). As such, restoring the E/I balance in neuronal networks through chloride homeostasis regulation might be a promising treatment target to alleviate behavioral symptoms in TSC such as irritability, social, and repetitive behavior (Ben-Ari, 2017; Vlaskamp et al., 2017; van Andel et al., 2020).

Bumetanide, a safe diuretic drug candidate for ASD and epilepsy treatment (Kahle et al., 2009; Eftekhari et al., 2013; Lemonnier et al., 2017; Gharaylou et al., 2019; Kharod et al., 2019; Sprengers et al., 2020), is a selective antagonist of NKCC1. This agent may thus influence E/I-balance regulation *via* downregulation of intraneuronal chloride concentration, which in turn can shift GABA polarity from depolarizing (with excitatory activity) to hyperpolarizing (inhibitory activity) (Löscher et al., 2013; Ben-Ari, 2017; Kharod et al., 2019). Through this mechanism, it is hypothesized that bumetanide may be beneficial for neurodevelopmental disorders (e.g., epilepsy, ASD, and TSC) (Talos et al., 2012; Ben-Ari, 2017; Kharod et al., 2019; van Andel et al., 2020; Juarez-Martinez et al., 2021). In animal models, it has indeed been shown that bumetanide can reduce the frequency of epileptiform activity (Dzhala et al., 2008; Marguet et al., 2015; Auer et al., 2020). In a clinical setting, cases on reduction of seizure frequency after bumetanide treatment have been reported (Eftekhari et al., 2013; Gharaylou et al., 2019). Bumetanide also seemed to effectively revert GABA-mediated excitatory effects in human TSC dysplastic cortex (*in vitro*) (Talos et al., 2012). Importantly, in our open-label trial in children and adolescents with TSC, our group recently reported improvement in irritability, hyperactivity, social behavior, and quality of life after bumetanide treatment, although no changes in seizure frequency were noted (van Andel et al., 2020). In two other genetic models of ASD, bumetanide decreased neuronal chloride concentrations and restored GABA inhibitory activity, improving behavior (Tyzio et al., 2014). Furthermore, we and others showed beneficial effects of bumetanide on core symptomatology in children and adolescents with ASD (Lemonnier et al., 2012, 2017; Sprengers et al., 2020; Zhang et al., 2020), such as social responsiveness (Lemonnier et al., 2017) and repetitive behavior (Sprengers et al., 2020).

These neurological effects of bumetanide and their relation with clinical improvement are, however, disputed given its limited crossing of the blood-brain barrier and poor brain bioavailability (Puskarjov et al., 2014; Kharod et al., 2019). EEG measures that are sensitive to E/I-balance modulation may clarify the neurophysiological effects of bumetanide. Indeed, EEG measures are widely used to diagnose and monitor treatment effects in epilepsy. More recently, quantitative EEG has been increasingly used for monitoring neuropsychiatric disorders, including depression (Rajpurkar et al., 2020; Wu et al., 2020; Zhdanov et al., 2020) and in ASD trials (Wang et al., 2013; Jeste et al., 2015; Heunis et al., 2016). For instance, we and others have shown that functional E/I ratios (*f*E/I) in neuronal networks may be quantified using EEG (Bruining et al., 2020; Donoghue et al., 2020). *f*E/I, together with other measures of critical brain dynamics, such as power and long-range temporal correlations (LRTC), have shown sensitivity to E/I changes in disorders such as ASD and epilepsy, and responsive to pharmacological intervention (Monto et al., 2007; Smith et al., 2017; Bruining et al., 2020). Importantly, using this novel resting-state EEG analysis, we recently confirmed placebo-controlled neurophysiological effects of bumetanide in a cohort with ASD (Juarez-Martinez et al., 2021). Here, we used these measures to investigate network-level E/I imbalances in TSC before and after bumetanide treatment, hypothesizing that TSC would show a reduced inhibitory activity, which could be restored by bumetanide. Then we investigated the relationship between bumetanide’s neurophysiological effects and clinical outcomes.

## Methods

### Study Design and Participants

This study is a secondary analysis of the Bumetanide to Ameliorate Tuberous Sclerosis Complex Hyperexcitable Behaviors trial (BATSCH trial; Eudra-CT 2016-002408-13), a single-center, open-label pilot study testing the effect of bumetanide (twice-daily up to 1.0 mg) in patients with TSC. Detailed description of the protocol and clinical effects have been published previously (van Andel et al., 2020). Briefly, participants were recruited from March 2017 to April 2018 at the UMC Utrecht, Netherlands. The trial included participants aged 8–21 years with a definite TSC diagnosis (genetic or clinical according to the 2012 International Tuberous Sclerosis Complex Consensus Conference) (Northrup and Krueger, 2013). Intellectual disability and concomitant use of antiepileptic and psychoactive drugs were allowed. Outcomes were assessed at pretreatment (D0), after 91 days of treatment (D91), and after a 28-day washout period (D119). Clinical outcome measures included were the Aberrant Behavior Checklist-Irritability subscale (ABC-I; range 0-45, higher score is more affected) (Aman et al., 1985), Social Responsiveness Scale-2 (SR-2; total raw score; range 0-195; higher score indicates more affected) (Constantino et al., 2003), the Repetitive Behavior Scale-Revised (RBS-R; total raw score; range 0-129, higher score indicates more affected) (Lam and Aman, 2007) and resting-state EEG recordings.

EEG recordings from typically developing children (TDC) collected between 2015 and 2018 at the Department of Psychiatry, UMC Utrecht were also included to compare their measures against the pretreatment TSC sample. The TDC group (7–16 years old and IQ > 55) had no history of medical, developmental or learning problems and were medication-free. The study was approved by the medical ethical committee of the UMC Utrecht and conducted in accordance with the provisions of the Declaration of Helsinki. All participants or their legal guardians signed informed consent.

### EEG Recordings and Pre-processing

EEGs for the TSC and TDC samples were recorded in the morning during 5 min of eyes-closed rest (in a quiet EEG room) with a 64-channel BioSemi EEG system (sampling rate 2,048 Hz). EEG analyses were carried out using the Neurophysiological Biomarker Toolbox^1^ and custom-made scripts (Hardstone et al., 2012). All recordings were manually cleaned for artifacts and re-referenced to the average reference. After pre-processing, on average 234 s per recording (140–302 s) were available for analysis for the TSC sample. For the TDC sample, on average 242 s per recording (156–307 s) were available for analysis.

### EEG Analysis

We focused on alpha-band oscillations (8–13 Hz) due to their relevance for healthy neuronal network development and cognitive function (Palva and Palva, 2007) and their clear disruption in neurodevelopmental disorders (Wang et al., 2013; Dickinson et al., 2019). Computational neuronal network models generating alpha-band oscillations have shown that measures such as amplitude, frequency, temporal correlations, and *f*E/I are sensitive to changes in excitation/inhibition ratios (Poil et al., 2020; Bruining et al., 2020). Specifically, network oscillations have shown an increasing E/I ratio when the amplitude increases, whereas the temporal complexity of amplitude fluctuations – as reflected in long-range temporal auto-correlations (LRTC) – peak in an E/I-balanced network (Linkenkaer-Hansen et al., 2001; Hardstone et al., 2012). Additionally, a novel functional measure of E/I ratio (*f*E/I) showed responsivity to pharmacological intervention and a large heterogeneity in an ASD sample (Bruining et al., 2020). We hypothesized that these EEG measures could capture the neurophysiological changes induced by bumetanide in the TSC sample and would relate to the behavioral changes after treatment (Poil et al., 2020; Bruining et al., 2020).

#### Power and Frequency

A FIR-filter was applied to the EEG signal to extract the alpha-frequency band (8–13 Hz). Spectral power was computed using the Welch method with an 8192-point Blackman window and a frequency resolution of 0.12 Hz.

#### Temporal Structure

Neuronal oscillations exhibit complex fluctuations in amplitude that are characterized by a power-law decay of auto-correlations, also known as LRTC (Linkenkaer-Hansen et al., 2001). Computational modeling has shown that the strength of LRTC is influenced by the balance between excitatory and inhibitory signaling in the network producing the oscillations (Poil et al., 2020). We used DFA to quantify LRTC in the amplitude modulation of alpha oscillations in the time range of seconds to tens of seconds (Peng et al., 1995). The amplitude envelope of the alpha oscillations was extracted using the Hilbert transform applied to the FIR-filtered signals in the 8–13 Hz band. The analytical steps to quantify LRTC using DFA have been explained in detail previously (Linkenkaer-Hansen et al., 2001; Hardstone et al., 2012). Here, we used a standard assessment of the strength of LRTC on time scales from 2 to 20 s, which is reflected in the so-called “DFA exponent.” An exponent of 0.5 characterizes an uncorrelated signal whereas an exponent in the interval of 0.5–1.0 indicate LRTC with larger exponents indicating stronger correlations.

#### Functional Excitation/Inhibition Ratio

The algorithm for estimating E/I ratio was derived from the Critical Oscillations (CROS) model of ongoing neuronal activity (Poil et al., 2020), where strong associations are observed between the structural excitation/inhibition ratios, oscillation amplitude, and LRTC. From these observations, a functional form of E/I ratio (*fE*/*I*) can be estimated from the covariance of amplitude and LRTC within a signal (Bruining et al., 2020). This method was validated using pharmacological manipulation in healthy subjects using a GABAergic drug (zolpidem), corroborating a reduction of *f*E/I ratios after the drug administration, and already tested in an ASD sample supporting the notion of large heterogeneity in E/I ratios (for details see Bruining et al., 2020). The analytical steps for the algorithm are as follows: to test the relationship between the amplitude and LRTC of the amplitude envelope of an oscillatory signal, it is necessary to have a measure of LRTC on short time-scales that is unbiased by the amplitude of the signal. To this end, an amplitude-normalized fluctuation function, *nF*(*t*), is calculated as follows: The signal is band-pass filtered (*i*), the amplitude envelope *A* extracted (*ii*), the signal profile, *S* can then be calculated as the cumulative sum of the demeaned amplitude envelope (*iii*),


(1) $$S\left(t\right)=\sum_{k=1}^{t}\left(A\left(k\right)-\left\langle A\right\rangle \right)$$

and split into windows of a certain size (e.g., 5 s) in exactly the same way as during the DFA calculation (Peng et al., 1995; Hardstone et al., 2012). As an additional step (*iv*), each of these signal-profile windows is divided by the mean of the amplitude envelope for that window calculated during step (*ii*). These amplitude-normalized windows are then detrended (*v*) and, subsequently, the normalized fluctuation function is calculated for each window as the standard deviation of the amplitude-normalized signal profile (*vi*). To calculate the functional excitation/inhibition ratio, *fE*/*I*, Pearson correlation between the amplitude and the normalized fluctuation function for the set of windows *W* (*vii*) is performed. *fE*/*I* is then defined as:


(2) $$fE/I=1-r_{W_{amp},W_{nF\left(t\right)}}$$

Sub-critical (inhibition-dominated) networks are characterized by an *fE*/*I* < 1, super-critical (excitation-dominated) networks *fE*/*I* > 1, and critical (E/I-balanced) networks will have *fE*/*I* = 1. A DFA > 0.6 inclusion criterion for networks or channels before computing the *fE*/*I* is used because networks without LRTC will not show a co-variation of amplitude and the fluctuation function. For our analyses, *fE*/*I* was calculated for windows of 5 s with 80% overlap. Additional details can be found in Bruining et al. (2020).

### Statistical Analysis

Whole-brain average EEG biomarker values were compared between children with TSC (pre-treatment) and TDC using a *t*-test or Wilcoxon rank-sum test (depending on whether normality assumptions were met). Additionally, an ANCOVA test was performed to control for age and IQ as covariates. Six brain regions were designated to localize areas with significant differences among groups, and to avoid an excessive number of comparisons within our small sample size if we were to analyze individual EEG channels. The six regions were defined by the following channels: frontal (FP1, AF7, AF3, F1, F3, FPZ, AFZ, FZ, FP2, AF8, AF4, F2, and F4), temporal left (F5, F7, FC5, FT7, C5, T7, CP5, and TP7), temporal right (F6, FC6, C6, CP6, F8, FT8, T8, and TP8), central (FC1, FC3, C1, C3, FCZ, CZ, FC2, FC4, C2, C4, CP1, CP3, CP2, CP4, and CPZ), parietal (P1, P3, P5, P7, P9, PZ, P2, P4, P6, P8, and P10), and occipital (PO7, PO3, O1, POZ, OZ, PO8, PO4, O2, and IZ). Brain-region comparisons between groups were also performed using a *t*-test or Wilcoxon rank-sum test.

To assess whole-brain treatment effect of bumetanide on EEG measures, we compared the average biomarker value (mean) of the 64 channels at each timepoint using a paired *t*-test. Effect sizes for significant effects were calculated using Cohen’s *d*. The distribution of bumetanide EEG effects was also analyzed by comparing each region at different timepoints. Finally, we investigated the correlation between clinical outcome scales and EEG measures using Pearson or Spearman correlation coefficient (depending on whether normality assumptions were met). False discovery rate (FDR; *q* = 0.15) was used to correct for multiple testing at region level. We determined the *q* level of FDR by calculating the number of regions that would be false discoveries for different values of *q*. A *q* = 0.05 would allow no false discoveries if all six regions came out significant, which we considered too stringent. A *q* = 0.15 would allow for one region to be a false discovery if all six regions came out significant. Significance level was set at *p* < 0.05.

## Results

**FIGURE 1:**
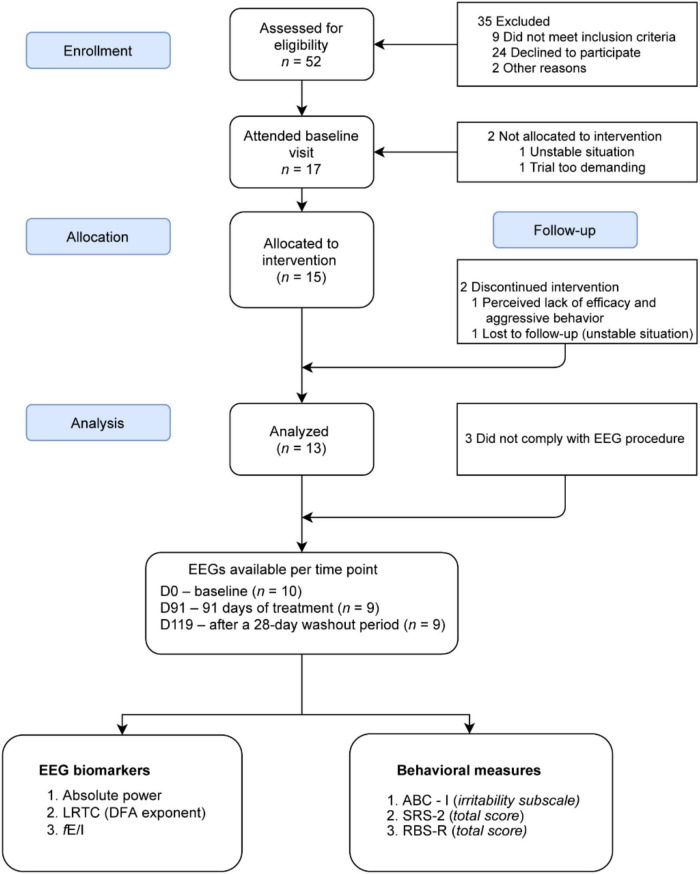
CONSORT flow diagram for the patients included for EEG analysis from the BATSCH trial. A total of 52 subjects were screened for eligibility from March 2017 to April 2018 at the UMC Utrecht, Netherlands. 15 participants received bumetanide. EEG recordings of 10 participants were available for analysis. CONSORT, Consolidated Standards of Reporting Trials; BATSCH, Bumetanide to Ameliorate Tuberous Sclerosis Complex Hyperexcitable Behaviors trial; ABC-I, Aberrant Behavior Checklist-Irritability subscale; SRS-2, Social Responsiveness Scale-2; RBS-R, Repetitive Behavior Scale-Revised; LRTC, long-range temporal correlations; DFA, scaling exponent of the detrended fluctuation analysis; *fE*/*I*, excitation-inhibition ratio.

Fifteen participants with TSC were allocated to bumetanide intervention of which 13 completed the trial (see van Andel et al., 2020 for cohort description and sample characteristics and Figure 1). EEG recordings of 10 participants were available for analysis (8.3–21.3 years, *M* = 13.4 ± 4 years, 5 females), 10 EEGs at D0, 9 at D91, and 9 at D119. Supplementary Tables 1, 2 describe the participants included, their demographics, clinical scores, and EEG biomarker values. EEG recordings of 29 TDC were included for baseline comparisons (7.4–14.4 years, *M* = 10.3 ± 1.5 years, 15 females). The mean age in the TSC group was 3.1 years higher than the TDC group (*p* = 0.04) and the total IQ was lower in TSC (*p* = 0.0001) (Table 1). Three EEG measures in the alpha band sensitive to changes in E/I-balance were computed (see section “Methods”).

**TABLE 1:** Clinical characteristics of participants with TSC and typically developing children.

	TDC	TSC	*t* (df)/*W*, *z*	*p-*Value
*n*	29	10	–	–
Males/females	14/15	5/5	–	–
Age (mean ± SD)	10.3 ± 1.5	13.4 ± 4	−2.4 (10)	0.04
TIQ (mean ± SEM)	120 ± 2.6	76 ± 7.05	5.8 (11)	0.0001
ABC-I (mean ± SEM)	1 ± 0.3	12.2 ± 2.2	329, 4.6	<0.0001
SRS-2 (mean ± SEM)	17.7 ± 1.5	74 ± 8.6	−6.4 (9)	0.0001
RBS-R (mean ± SEM)	0.8 ± 0.03	13.8 ± 4.8	313, 4.2	<0.0001

*Mean values and comparison’s statistics [t-test for parametric data (t, df) and Wilcoxon rank-sum test for non-parametric data (W, z). Demographics and EEG mean biomarker values per subject included in our study can be found on Supplementary Table 1.*

*TIQ, Total Intelligence Quotient; ABC-I, Aberrant Behavioral Checklist-Irritability subscale; SRS-2, Social Responsiveness Scale-2; RBS-R, Repetitive Behavioral Scale-Revised.*

### Spectral, Temporal, and Functional Excitation-Inhibition Ratio Characteristics of EEG in Tuberous Sclerosis Complex

**FIGURE 2:**
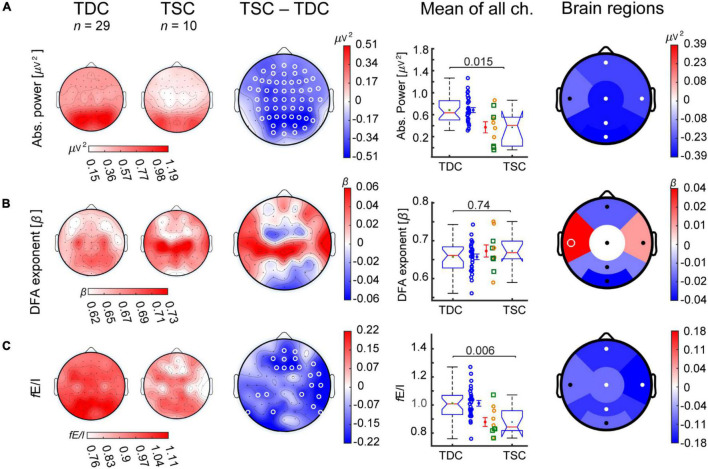
Participants with TSC show lower alpha-band absolute power and *f*E/I than TDC. Grand-average topographies are shown for TDC (*first column*), TSC (D0; *second column*), and for TSC-minus-TDC (*third column*) for the alpha-band biomarkers **(A)** absolute power, **(B)** DFA, and **(C)** *f*E/I. **(A)** The TSC group (D0, baseline) showed lower absolute power compared to TDC in most electrode locations and regions. **(B)** The DFA exponent was not different between groups. **(C)** *f*E/I ratios were also lower in TSC compared to TDC, particularly in frontal and temporal right regions. Comparisons in boxplots (*fourth column*) were based on the average value of the EEG biomarkers across all 64 channels (Wilcoxon rank-sum test). Individual-subject values, mean, and SEM are shown for TDC (*blue circles*) and TSC [concomitant treatment with antiepileptic drug (*yellow circles*) or not (*green squares*)]. Comparisons between groups are also shown for regional averages of frontal, temporal left, temporal right, central, parietal, and occipital electrodes (*fifth column*). White circles represent significant regions (*p*-value < 0.05, Wilcoxon rank-sum test, FDR corrected).

We first investigated spectral, temporal, and functional excitation-inhibition ratio (*f*E/I) characteristics of alpha oscillations at baseline in TSC in comparison to TDC. The TSC group showed widespread lower absolute power and *f*E/I ratios compared to TDC (Figures 2A,C). Both measures were significant at the level of whole-brain average, with absolute power being significant in five out of the six regions (frontal, central, temporal right, parietal, and occipital) and *f*E/I in four out of the six regions (frontal, central, temporal right, and parietal) (Figure 2 and Supplementary Figure 1). DFA did not show significant differences between groups in any regions (Figure 2B).

To explore the contribution of age and IQ to these EEG differences at whole-brain level, we additionally performed an ANCOVA test. After adjustment for age, a significant *f*E/I difference between TSC and TDC remained [*F*(1,36) = 6.79, *p* = 0.01, partial η^2^ = 0.16], and the difference in absolute power became borderline significant [*F*(1,36) = 4.0, *p* = 0.05, partial η^2^ = 0.1]. After adjustment for IQ, differences in absolute power and *f*E/I became non-significant [*F*(1,35) = 2.9, *p* = 0.09, partial η^2^ = 0.07 and *F*(1,35) = 2.1, *p* = 0.15, partial η^2^ = 0.06 respectively].

### Bumetanide Increased Absolute Power in Tuberous Sclerosis Complex

**FIGURE 3:**
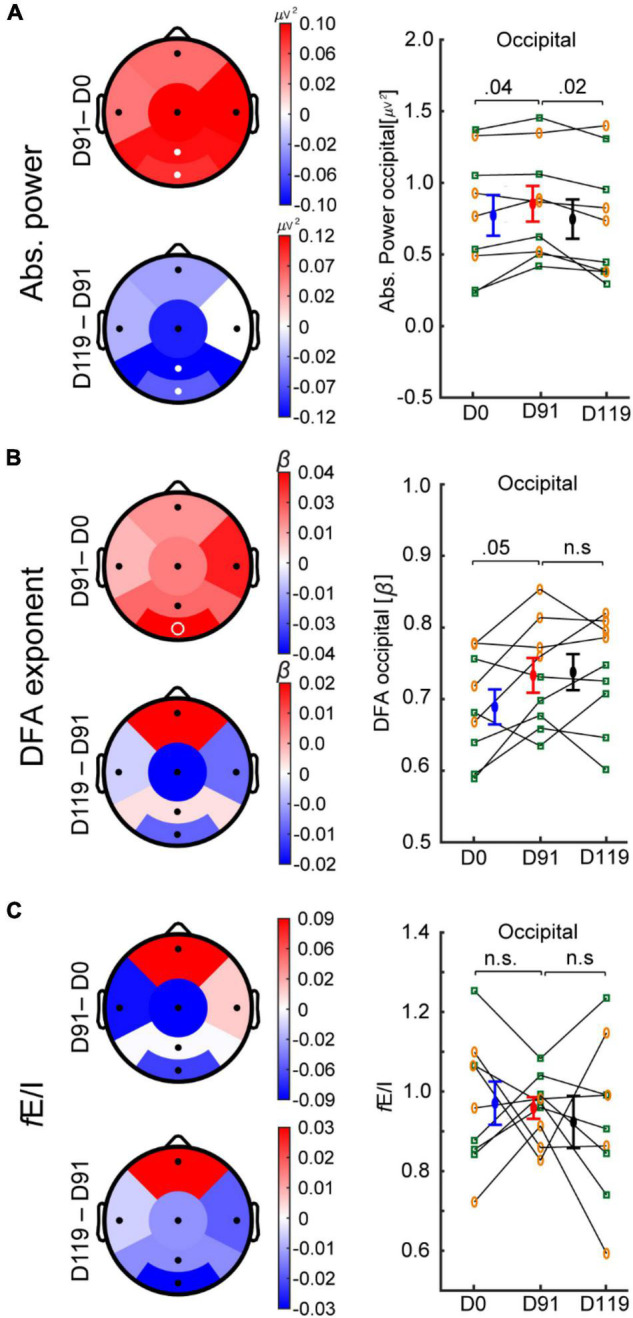
Bumetanide treatment increases the power of alpha oscillations in TSC. Treatment effects on EEG measures in six regions (D91-minus-D0 and D119-minus-D91) for absolute power, DFA, and *f*E/I are shown. **(A)** After 91 days of bumetanide treatment, absolute power increased in parietal and occipital regions, which returned to baseline after the washout period. **(B)** DFA increased in the occipital region, but did not reach significance. **(C)** *f*E/I mean values were not different between time points. Bumetanide appeared to reduce the *f*E/I variance in occipital region (Bartlett test of variance *p* = 0.06), which increased after the washout period (Bartlett *p* = 0.02). White filled circles represent significant regions (paired *t*-test, *p*-value < 0.05, FDR corrected), white unfilled circles indicate *p* < 0.05 without FDR correction. Individual values, mean, and SEM are plotted per time point for the occipital region **(A–C)**. Concomitant treatment with antiepileptic drug (*yellow circles*) or not (*green squares*). D0, day-zero baseline recording; D91, day 91 of treatment; D119, day 119 (after 28-days of washout period).

After 91 days of bumetanide treatment, only regional effects on the EEG were observed. Specifically, the absolute power increased in parietal and occipital regions (Figure 3A; Cohen’s *d* = 0.3 and 0.2, respectively). After the washout period (D119) absolute power decreased in the parietal region (Cohen’s *d* = 0.4) and the occipital region (Cohen’s *d* = 0.3). LRTC increased in the occipital region; however, this was not significant after multiple-comparison correction (Figure 3B, *p* = 0.05). We did not find a significant change in *f*E/I in any regions (Figure 3C).

### Correlation Between EEG Measures and Clinical Outcomes

**FIGURE 4:**
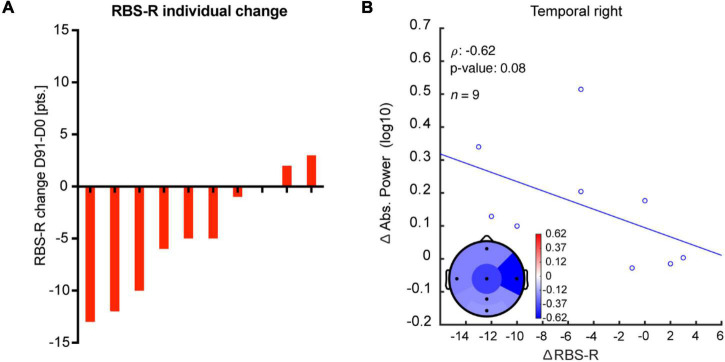
Change in absolute power after bumetanide shows a moderate, albeit non-significant, correlation with improvement in repetitive behavior. **(A)** Rank-ordered change in RBS-R in response to bumetanide for each participant (D91-minus-D0). **(B)** A moderate correlation, yet non-significant, was observed between an increment in absolute power and improvement in repetitive behavior after bumetanide (RBS-R), specifically in the right temporal region.

To test the clinical significance of the EEG effects of bumetanide, we analyzed the relationship between the change in EEG measures and clinical outcomes. Although we observed a moderate correlation between the increment in absolute power in the right temporal region and improvement in repetitive behavior (RBS-R) after bumetanide (D91-D0 change), this was not significant (*rho* = −0.6, *p* = 0.08; Figure 4). No regional associations were found between change in LRTC or *f*E/I and clinical outcomes.

**FIGURE 5:**
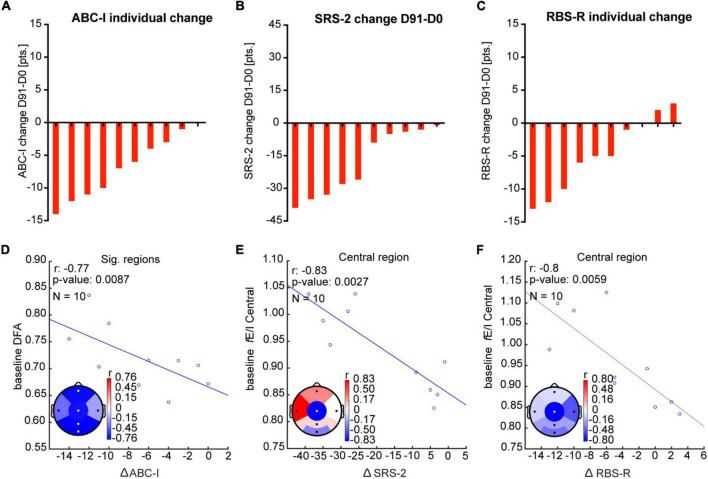
Baseline EEG measures correlate with clinical outcomes after bumetanide. **(A–C)** Rank-ordered change in ABC-I, SRS-2, and RBS-R in response to bumetanide for each participant (D91-minus-D0). **(D)** Improvement in irritability score (ABC-I) was strongly related (*rho* = 0.8) to higher DFA at baseline, particularly in frontal, central, parietal, and occipital regions. **(E,F)**. We found a strong correlation (*rho* = 0.8) between high *f*E/I at baseline in the central region and improvement in SRS-2 and RBS-R. Significant regions are represented with white dots (Pearson correlation coefficient, *p* < 0.05, FDR corrected *q* = 0.15).

Baseline neurophysiological characteristics (i.e., before treatment), such as E/I setpoints, might influence the response to bumetanide. To test this idea, we investigated the relationship between baseline EEG characteristics and clinical outcomes (Figure 5). We found that high LRTC at baseline correlated strongly to improvement in irritability symptoms in frontal, central, parietal, and occipital regions (ABC-I score; *rho* = −0.8, *p* = 0.009; Figure 5D). We did not find correlations between baseline absolute power nor *f*E/I and ABC-I outcomes. High *f*E/I at baseline in the central region correlated strongly to improvement in social response (SRS-2; *rho* = −0.8, *p* = 0.003) and repetitive behavior (RBS-R; *rho* = −0.8, *p* = 0.006; Figures 5E,F). No correlations were found between baseline absolute power nor LRTC for SRS-2 or RBS-R scores.

## Discussion

The results of this secondary resting-state EEG analysis in TSC showed aberrant network E/I balance, reflected in lower alpha power and *f*E/I. EEG measures sensitive to E/I changes were modified through bumetanide treatment; however, the correlation between these EEG changes and clinical improvement was inconclusive. In contrast, we found that pretreatment EEG characteristics were associated with clinical improvement for all three clinical scales, implying that baseline network features might influence treatment response. Together, these findings highlight the utility of E/I-sensitive EEG measures to accompany new treatment interventions for TSC.

### Network-Level Excitation-Inhibition Imbalances in Tuberous Sclerosis Complex

Evidence of perturbed neuronal E/I balance in TSC has been described at different levels, from decreased number and development of GABAergic interneurons to abnormal excitatory GABA responses linked to aberrant chloride homeostasis (Fu et al., 2012; Talos et al., 2012; Ruffolo et al., 2016). Using three EEG measures sensitive to changes in E/I, we show that network-level E/I balance is altered in TSC. We found lower alpha-band power in TSC compared to TDC—an observation that has been described also in ASD (Kulandaivel and Holmes, 2011; Tierney et al., 2012; Wang et al., 2013; Bruining et al., 2020) and in attention deficit and hyperactivity disorder (Deiber et al., 2020). Furthermore, in TSC studies, it has been associated with delayed cognitive and motor development (Dickinson et al., 2019; De Ridder et al., 2021). Albeit the link between network-level E/I balance and alpha power remains uncertain, computational models have related decreasing alpha power to increasing inhibitory connections or inhibitory signaling (Poil et al., 2020; Bruining et al., 2020).

Contrary to what we expected in TSC, we observed that the functional E/I ratio (*f*E/I) was below 1.0, and lower compared to the TDC. This finding might seem counterintuitive given the preclinical and clinical evidence of reduced inhibition in TSC (White et al., 2001; Wang et al., 2007; Taki et al., 2009; Fu et al., 2012; Mori et al., 2012; Talos et al., 2012; Bateup et al., 2013; Aronica and Crino, 2014; Ruffolo et al., 2016; Nadadhur et al., 2019; Afshar Saber and Sahin, 2020; Haji et al., 2020) and the high epilepsy comorbidity in this group. In principle, the use of antiepileptic drugs (AED) that enhance inhibitory signaling could have contributed to the lower *f*E/I, but the lowest *f*E/I values were found amongst the subjects that were not taking AEDs. Thus, we speculate that the presence of inhibition-dominated networks in TSC may relate to neurodevelopmental delay and cortical dysfunction (e.g., due to cortical tubers and aberrant surrounding tissue). Indeed, signs of cortical dysfunction, dysmaturity, and epilepsy [e.g., as reflected by slowing of activity (Krsek et al., 2010; Terney et al., 2010; Tao et al., 2011; Stoner et al., 2014; Britton et al., 2016)] have been described in EEG recordings in TSC (De Ridder et al., 2020). Importantly, we have previously associated EEG abnormalities to low *f*E/I in ASD (Bruining et al., 2020) and in children with STXBP1 syndrome, which is another genetic disorder with a high prevalence of epilepsy (Houtman et al., 2021). Furthermore, low *f*E/I values in TSC could be an expression of both, an excess in inhibition, e.g., as a protective mechanism against epileptogenicity (Terney et al., 2010; Bruining et al., 2020), increased GABA concentration (i.e., in cortical tubers) compensating for reduced GABA receptors (Taki et al., 2009; Mori et al., 2012), or an overall reduced excitation as a compensatory change for a primary aberrant (excitatory) GABA transmission (Nelson and Valakh, 2015). Overall, these all suggest that network-level E/I balance might be different from that seen at a neuronal or synapse level, likely due to more complex network reorganization or overriding compensatory mechanisms.

In sum, we found aberrant dynamics of neuronal oscillations in TSC pointing toward an inhibition-dominated network. Importantly, the similarities in alpha power and *f*E/I characteristics between our TSC sample and what has been reported in other developmental disorders (e.g., ASD), suggest a link between altered network E/I activity and aberrant behavior.

### EEG Effects of Bumetanide in Tuberous Sclerosis Complex

We next tested the effect of bumetanide on EEG measures sensitive to E/I changes and investigated the relationship of treatment effects with clinical outcomes. Bumetanide increased the power of alpha oscillations after three months of treatment, an effect that showed a moderate, yet non-significant, correlation with improvement in repetitive behavior, specifically, for the temporal region [a region that has shown relevance for stereotypes and repetitive behavior in an ASD context (Ohnishi et al., 2000; Boddaert et al., 2004; Gendry Meresse et al., 2005)]. A case report in TSC from our group had previously described similar effects of bumetanide on alpha power with beneficial behavioral outcomes (Vlaskamp et al., 2017). It is unclear whether this effect implies restoring the characteristics of alpha oscillations, but it appears to be favorable in neurodevelopmental disorders. For instance, in epilepsy, an increment in alpha power following AEDs (e.g., levetiracetam) has been associated with improvement in neuropsychological tests (Cho et al., 2012; Ricci et al., 2021). Similarly, we reported a relationship between increased alpha power and improvement in repetitive behavior after bumetanide in children with ASD (Juarez-Martinez et al., 2021). Interestingly, the EEG effect of bumetanide is contrary to what has been described for other GABAergic drugs (benzodiazepines) in healthy subjects (i.e., a reduction in alpha power) (Lozano-Soldevilla et al., 2014; Lozano-Soldevilla, 2018). This calls into question whether GABA-modulating agents might have a divergent network repercussion when administered in a healthy vs. diseased brain since in the latter reorganization of the network might have taken place or compensatory mechanisms may be taking over. In sum, our results suggest that bumetanide may induce a neurophysiological effect in TSC, that can potentially translate into behavioral improvement, specifically for repetitive behavior. However, investigations in a larger sample are necessary to support the clinical significance of bumetanide’s effect.

### Baseline EEG Characteristics Might Be Associated With Clinical Improvement

Significant improvement in social response and irritability were described after bumetanide in the original paper (van Andel et al., 2020). However, we did not find a relationship between a neurophysiological change after treatment and improvement in these clinical outcomes. Variability in network characteristics may account for differences in treatment response in neurodevelopmental disorders. Indeed, large variability in LRTC and *f*E/I have been proposed to contribute to the physiological heterogeneity in ASD (Bruining et al., 2020). Importantly, we reported that baseline EEG features in ASD may have a predictive value for clinical improvement (e.g., after bumetanide treatment) (Juarez-Martinez et al., 2021). Thus, baseline network characteristics in TSC may indicate different “network setpoints” in which bumetanide may induce clinically meaningful effects. Here, we observed that high LRTC at baseline was strongly related to improvement in irritability (ABC-I) after treatment. LRTC can be an indicator of network adaptability, where high values indicate a more readily adaptable structure, thus may be more easily influenced by bumetanide. This is not an entirely unexpected finding. LRTC are sensitive to AEDs, which were commonly used in our sample. AEDs have shown to increase LRTC in epilepsy (Smith et al., 2017), thus it is plausible that the initially high LRTC was a primary effect of the AEDs, and the beneficial effects of higher LRTC (through treatment) were further boosted by bumetanide co-medication. Interestingly, computational models have shown that LRTC peak when the network exhibits a balanced E/I (Bruining et al., 2020). Here, high *f*E/I at baseline strongly related to improvement in social response and repetitive behavior, where the greater improvement was seen in those with *f*E/I closer to or above 1 (mainly in the central region). We can argue that networks closer to the “balanced” point at baseline (either through compensatory mechanisms or as an AED effect), more readily achieve optimal network dynamics through relatively small changes induced by bumetanide (possibly as an add-on medication), improving behavior.

### Limitations

Important limitations of this study are the small sample size, the open-label design of the study and the lack of a placebo group to compare the effects of bumetanide on resting-state EEG. TSC is a rare genetic disorder and its comorbidities may pose a major burden and disability which makes recruiting a large number of participants challenging, even more given the rigorous demands that a placebo-controlled trial would entail. However, we did include EEGs of TDC (albeit with a narrower age range) to compare the baseline characteristics of our TSC sample. Age difference between groups did not significantly impact our results, however, IQ did. Yet, it is important to clarify that IQ difference itself is an essential component in neurodevelopmental disorders rather than a confounding factor (Dennis et al., 2009). Another limitation is the concomitant administration of antiepileptic medication (AEDs). Although the AED dosage was kept stable throughout the study, it still limits the interpretation of EEG measures that are sensitive to such drugs. Additionally, regular use of AEDs could have masked bumetanide EEG effects. Given the small sample size, we limited our analysis to only alpha frequency band and only three clinical scales. It is possible that alterations in other frequency bands (e.g., theta, beta, and gamma) might have a relevant impact on behavioral outcomes and might be more sensitive to bumetanide effects. Future studies investigating the role of neuronal oscillations other than alpha in TSC are necessary, as well as corroborating our findings in a larger group sample.

## Conclusion

Our findings showed that network-level E/I imbalances are present in TSC pointing toward an abundance of inhibition-dominated networks. Bumetanide induced a neurophysiological effect in TSC, and we show that baseline network characteristics might influence treatment response. These findings highlight the possible utility of E/I-sensitive EEG measures to accompany new treatment interventions for TSC.

## Data Availability Statement

The original contributions presented in the study are included in the article/Supplementary Material, further inquiries can be directed to the corresponding author.

## Ethics Statement

The studies involving human participants were reviewed and approved by the Medical Ethical Committee of the University Medical Center (UMC) Utrecht. Written informed consent to participate in this study was provided by the participants’ legal guardian/next of kin.

## Author Contributions

HB and KL-H: conceptualization and funding acquisition. EJ-M, DA, JS, KL-H, and HB: methodology and writing—original draft. EJ-M, DA, and JS: investigation. EJ-M and A-EA: visualization. A-EA, BO, and FJ: administrative, technical, or material support. HM, FS, KL-H, and HB: supervision. All authors contributed to acquisition, analysis, or interpretation of data, and writing—reviewing and editing.

## Conflict of Interest

KL-H is shareholder of NBT Analytics BV, which provides EEG-analysis services for clinical trials. HB and KL-H are shareholders of Aspect Neuroprofiles BV, which develops physiology-informed prognostic measures for neurodevelopmental disorders. KL-H has filed the patent claim (PCT/NL2019/050167) “Method of determining brain activity”; with priority date 16 March 2018. The remaining authors declare that the research was conducted in the absence of any commercial or financial relationships that could be construed as a potential conflict of interest.

## Publisher’s Note

All claims expressed in this article are solely those of the authors and do not necessarily represent those of their affiliated organizations, or those of the publisher, the editors and the reviewers. Any product that may be evaluated in this article, or claim that may be made by its manufacturer, is not guaranteed or endorsed by the publisher.

## Footnotes

1
http://www.nbtwiki.net/

## References

[B1] Afshar Saber W. Sahin M. (2020). Recent advances in human stem cell-based modeling of tuberous sclerosis complex. *Mol. Autism.* 11:16. 10.1186/s13229-020-0320-2 32075691 PMC7031912

[B2] Aman M. G. Singh N. N. Stewart A. W. Field C. J. (1985). The aberrant behavior checklist: a behavior rating scale for the assessment of treatment effects. *Am. J. Ment. Defic.* 89 485–491. 3993694

[B3] Aronica E. Crino P. B. (2014). Epilepsy related to developmental tumors and malformations of cortical development. *Neurotherapeutics* 11 251–268. 10.1007/s13311-013-0251-0 24481729 PMC3996119

[B4] Auer T. Schreppel P. Erker T. Schwarzer C. (2020). Functional characterization of novel bumetanide derivatives for epilepsy treatment. *Neuropharmacology* 162:107754. 10.1016/j.neuropharm.2019.107754 31476353

[B5] Avramiea A. E. Hardstone R. Lueckmann J. M. Bím J. Mansvelder H. D. Linkenkaer-Hansen K. (2020). Pre-stimulus phase and amplitude regulation of phase-locked responses are maximized in the critical state. *eLife* 9:e53016. 10.7554/eLife.53016 32324137 PMC7217696

[B6] Bateup H. S. Johnson C. A. Denefrio C. L. Saulnier J. L. Kornacker K. Sabatini B. L. (2013). Excitatory/inhibitory synaptic imbalance leads to hippocampal hyperexcitability in mouse models of tuberous sclerosis. *Neuron* 78 510–522. 10.1016/j.neuron.2013.03.017 23664616 PMC3690324

[B7] Ben-Ari Y. (2017). NKCC1 chloride importer antagonists attenuate many neurological and psychiatric disorders. *Trends Neurosci.* 40 536–554. 10.1016/j.tins.2017.07.001 28818303

[B8] Boddaert N. Chabane N. Gervais H. Good C. D. Bourgeois M. Plumet M. H. (2004). Superior temporal sulcus anatomical abnormalities in childhood autism: a voxel-based morphometry MRI study. *Neuroimage* 23 364–369. 10.1016/j.neuroimage.2004.06.016 15325384

[B9] Britton J. W. Frey L. C. Hopp J. L. Korb P. Koubeissi M. Z. Lievens W. E. (2016). *Electroencephalography (EEG): An Introductory Text and Atlas of Normal and Abnormal Findings in Adults, Children, and Infants.* Chicago, IL: American Epilepsy Society. 27748095

[B10] Bruining H. Hardstone R. Juarez-Martinez E. L. Sprengers J. Avramiea A. E. Simpraga S. (2020). Measurement of excitation-inhibition ratio in autism spectrum disorder using critical brain dynamics. *Sci. Rep.* 10:9195. 10.1038/s41598-020-65500-4 32513931 PMC7280527

[B11] Cho J. R. Koo D. L. Joo E. Y. Yoon S. M. Ju E. Lee J. (2012). Effect of levetiracetam monotherapy on background EEG activity and cognition in drug-naïve epilepsy patients. *Clin. Neurophysiol.* 123 883–891. 10.1016/j.clinph.2011.09.012 22000706

[B12] Constantino J. N. Davis S. A. Todd R. D. Schindler M. K. Gross M. M. Brophy S. L. (2003). Validation of a brief quantitative measure of autistic traits: comparison of the social responsiveness scale with the autism diagnostic interview-revised. *J. Autism Dev. Disord.* 33 427–433. 10.1023/a:1025014929212 12959421

[B13] Curatolo P. (2015). Mechanistic target of rapamycin (mTOR) in tuberous sclerosis complex-associated epilepsy. *Pediatr. Neurol.* 52 281–289. 10.1016/j.pediatrneurol.2014.10.028 25591831

[B14] Curatolo P. Bombardieri R. Jozwiak S. (2008). Tuberous sclerosis. *Lancet* 372 657–668. 18722871 10.1016/S0140-6736(08)61279-9

[B15] De Ridder J. Kotulska K. Curatolo P. Jansen A. C. Aronica E. Kwiatkowski D. J. (2021). Evolution of electroencephalogram in infants with tuberous sclerosis complex and neurodevelopmental outcome: a prospective cohort study. *Dev. Med. Child Neurol.* 64 495–501. 10.1111/dmcn.15073 34601720

[B16] De Ridder J. Lavanga M. Verhelle B. Vervisch J. Lemmens K. Kotulska K. (2020). Prediction of neurodevelopment in infants with tuberous sclerosis complex using Early EEG characteristics. *Front. Neurol.* 11:582891. 10.3389/fneur.2020.582891 33178126 PMC7596378

[B17] de Vries P. J. (2010). Targeted treatments for cognitive and neurodevelopmental disorders in tuberous sclerosis complex. *Neurotherapeutics* 7 275–282. 10.1016/j.nurt.2010.05.001 20643380 PMC5084231

[B18] de Vries P. J. Whittemore V. H. Leclezio L. Byars A. W. Dunn D. Ess K. C. (2015). Tuberous sclerosis associated neuropsychiatric disorders (TAND) and the TAND checklist. *Pediatr. Neurol.* 52 25–35. 10.1016/j.pediatrneurol.2014.10.004 25532776 PMC4427347

[B19] Deiber M. P. Hasler R. Colin J. Dayer A. Aubry J. M. Baggio S. (2020). Linking alpha oscillations, attention and inhibitory control in adult ADHD with EEG neurofeedback. *Neuroimage Clin.* 25:102145. 10.1016/j.nicl.2019.102145 31911342 PMC6948256

[B20] Dennis M. Francis D. J. Cirino P. T. Schachar R. Barnes M. A. Fletcher J. M. (2009). Why IQ is not a covariate in cognitive studies of neurodevelopmental disorders. *J. Int. Neuropsychol. Soc.* 15 331–343. 10.1017/S1355617709090481 19402919 PMC3075072

[B21] Dickinson A. Varcin K. J. Sahin M. Nelson C. A. III Jeste S. S. (2019). Early patterns of functional brain development associated with autism spectrum disorder in tuberous sclerosis complex. *Autism Res.* 12 1758–1773. 10.1002/aur.2193 31419043 PMC6898751

[B22] Donoghue T. Haller M. Peterson E. J. Varma P. Sebastian P. Gao R. (2020). Parameterizing neural power spectra into periodic and aperiodic components. *Nat. Neurosci.* 23 1655–1665. 10.1038/s41593-020-00744-x 33230329 PMC8106550

[B23] Dzhala V. I. Brumback A. C. Staley K. J. (2008). Bumetanide enhances phenobarbital efficacy in a neonatal seizure model. *Ann. Neurol.* 63 222–235. 10.1002/ana.21229 17918265

[B24] Eftekhari S. Mehvari Habibabadi J. Najafi Ziarani M. Hashemi Fesharaki S. S. Gharakhani M. Mostafavi H. (2013). Bumetanide reduces seizure frequency in patients with temporal lobe epilepsy. *Epilepsia* 54 e9–e12. 10.1111/j.1528-1167.2012.03654.x 23061490

[B25] Fu C. Cawthon B. Clinkscales W. Bruce A. Winzenburger P. Ess K. C. (2012). GABAergic interneuron development and function is modulated by the Tsc1 gene. *Cereb Cortex.* 22 2111–2119. 10.1093/cercor/bhr300 22021912 PMC3412444

[B26] Gendry Meresse I. Zilbovicius M. Boddaert N. Robel L. Philippe A. Sfaello I. (2005). Autism severity and temporal lobe functional abnormalities. *Ann. Neurol.* 58 466–469. 10.1002/ana.20597 16130096

[B27] Gharaylou Z. Tafakhori A. Agah E. Aghamollaii V. Kebriaeezadeh A. Hadjighassem M. (2019). A preliminary study evaluating the safety and efficacy of bumetanide, an NKCC1 inhibitor, in patients with drug-resistant epilepsy. *CNS Drugs* 33 283–291. 10.1007/s40263-019-00607-5 30784026

[B28] Haji N. Riebe I. Aguilar-Valles A. Artinian J. Laplante I. Lacaille J. C. (2020). Tsc1 haploinsufficiency in Nkx2.1 cells upregulates hippocampal interneuron mTORC1 activity, impairs pyramidal cell synaptic inhibition, and alters contextual fear discrimination and spatial working memory in mice. *Mol. Autism.* 11:29. 10.1186/s13229-020-00340-7 32375878 PMC7201610

[B29] Hardstone R. Poil S. S. Schiavone G. Jansen R. Nikulin V. V. Mansvelder H. D. (2012). Detrended fluctuation analysis: a scale-free view on neuronal oscillations. *Front. Physiol.* 3:450. 10.3389/fphys.2012.00450 23226132 PMC3510427

[B30] Heunis T. M. Aldrich C. de Vries P. J. (2016). Recent advances in resting-state electroencephalography biomarkers for autism spectrum disorder-a review of methodological and clinical challenges. *Pediatric. Neurol.* 61 28–37. 10.1016/j.pediatrneurol.2016.03.010 27255413

[B31] Houtman S. J. Lammertse H. C. A. van Berkel A. A. Balagura G. Gardella E. Ramautar J. R. (2021). STXBP1 syndrome is characterized by inhibition-dominated dynamics of resting-state EEG. *Front. Physiol.* 12:775172. 10.3389/fphys.2021.775172 35002760 PMC8733612

[B32] Jeste S. S. Frohlich J. Loo S. K. (2015). Electrophysiological biomarkers of diagnosis and outcome in neurodevelopmental disorders. *Curr. Opin. Neurol.* 28 110–116. 10.1097/WCO.0000000000000181 25710286 PMC7334029

[B33] Juarez-Martinez E. L. Sprengers J. J. Cristian G. Oranje B. van Andel D. M. Avramiea A. E. (2021). Prediction of behavioral improvement through resting-state EEG and clinical severity in a randomized controlled trial testing bumetanide in autism spectrum disorder. *Biol. Psychiatry Cogn. Neurosci. Neuroimaging.* Online ahead of print. 10.1016/j.bpsc.2021.08.009 34506972

[B34] Jülich K. Sahin M. (2014). Mechanism-based treatment in tuberous sclerosis complex. *Pediatr. Neurol.* 50 290–296. 10.1016/j.pediatrneurol.2013.12.002 24486221 PMC3959246

[B35] Kahle K. T. Barnett S. M. Sassower K. C. Staley K. J. (2009). Decreased seizure activity in a human neonate treated with bumetanide, an inhibitor of the Na(+)-K(+)-2Cl(-) cotransporter NKCC1. *J. Child Neurol.* 24 572–576. 10.1177/0883073809333526 19406757

[B36] Kharod S. C. Kang S. K. Kadam S. D. (2019). Off-Label use of bumetanide for brain disorders: an overview. *Front. Neurosci.* 13:310. 10.3389/fnins.2019.00310 31068771 PMC6491514

[B37] Kinouchi O. Copelli M. (2006). Optimal dynamical range of excitable networks at criticality. *Nat. Phys.* 2 348–351. 10.1038/nphys289

[B38] Krsek P. Jahodova A. Maton B. Jayakar P. Dean P. Korman B. (2010). Low-grade focal cortical dysplasia is associated with prenatal and perinatal brain injury. *Epilepsia* 51 2440–2448. 10.1111/j.1528-1167.2010.02730.x 20887366

[B39] Krueger D. A. Sadhwani A. Byars A. W. de Vries P. J. Franz D. N. Whittemore V. H. (2017). Everolimus for treatment of tuberous sclerosis complex-associated neuropsychiatric disorders. *Ann. Clin. Transl. Neurol.* 4 877–887. 10.1002/acn3.494 29296616 PMC5740257

[B40] Krueger D. A. Wilfong A. A. Holland-Bouley K. Anderson A. E. Agricola K. Tudor C. (2013). Everolimus treatment of refractory epilepsy in tuberous sclerosis complex. *Ann. Neurol.* 74 679–687. 10.1002/ana.23960 23798472

[B41] Kulandaivel K. Holmes G. L. (2011). Power spectral analysis in infants with seizures: relationship to development. *Epilepsy Behav.* 20 700–705. 10.1016/j.yebeh.2011.02.021 21439912

[B42] Lam K. S. Aman M. G. (2007). The repetitive behavior scale-revised: independent validation in individuals with autism spectrum disorders. *J. Autism Dev. Disord.* 37 855–866. 10.1007/s10803-006-0213-z 17048092

[B43] Leclezio L. de Vries P. J. (2015). Advances in the treatment of tuberous sclerosis complex. *Curr. Opin. Psychiatry.* 28 113–120. 25602245 10.1097/YCO.0000000000000136

[B44] Lemonnier E. Degrez C. Phelep M. Tyzio R. Josse F. Grandgeorge M. (2012). A randomised controlled trial of bumetanide in the treatment of autism in children. *Trans. Psychiatry* 2:e202. 10.1038/tp.2012.124 23233021 PMC3565189

[B45] Lemonnier E. Villeneuve N. Sonie S. Serret S. Rosier A. Roue M. (2017). Effects of bumetanide on neurobehavioral function in children and adolescents with autism spectrum disorders. *Transl. Psychiatry* 7:e1056. 10.1038/tp.2017.10 28291262 PMC5416661

[B46] Linkenkaer-Hansen K. Nikouline V. V. Palva J. M. Ilmoniemi R. J. (2001). Long-range temporal correlations and scaling behavior in human brain oscillations. *J. Neurosci.* 21 1370–1377. 10.1523/JNEUROSCI.21-04-01370.2001 11160408 PMC6762238

[B47] Löscher W. Puskarjov M. Kaila K. (2013). Cation-chloride cotransporters NKCC1 and KCC2 as potential targets for novel antiepileptic and antiepileptogenic treatments. *Neuropharmacology* 69 62–74. 10.1016/j.neuropharm.2012.05.045 22705273

[B48] Lozano-Soldevilla D. (2018). On the physiological modulation and potential mechanisms underlying parieto-occipital alpha oscillations. *Front. Comput. Neurosci.* 12:23. 10.3389/fncom.2018.00023 29670518 PMC5893851

[B49] Lozano-Soldevilla D. ter Huurne N. Cools R. Jensen O. (2014). GABAergic modulation of visual gamma and alpha oscillations and its consequences for working memory performance. *Curr. Biol.* 24 2878–2887. 10.1016/j.cub.2014.10.017 25454585

[B50] Marguet S. L. Le-Schulte V. T. Merseburg A. Neu A. Eichler R. Jakovcevski I. (2015). Treatment during a vulnerable developmental period rescues a genetic epilepsy. *Nat. Med.* 21 1436–1444. 10.1038/nm.3987 26594844

[B51] Monto S. Vanhatalo S. Holmes M. D. Palva J. M. (2007). Epileptogenic neocortical networks are revealed by abnormal temporal dynamics in seizure-free subdural EEG. *Cereb Cortex.* 17 1386–1393. 10.1093/cercor/bhl049 16908492

[B52] Mori K. Mori T. Toda Y. Fujii E. Miyazaki M. Harada M. (2012). Decreased benzodiazepine receptor and increased GABA level in cortical tubers in tuberous sclerosis complex. *Brain Dev.* 34 478–486. 10.1016/j.braindev.2011.09.001 21959128

[B53] Nadadhur A. G. Alsaqati M. Gasparotto L. Cornelissen-Steijger P. van Hugte E. Dooves S. (2019). Neuron-Glia interactions increase neuronal phenotypes in tuberous sclerosis complex patient iPSC-Derived models. *Stem Cell Reports.* 12 42–56. 10.1016/j.stemcr.2018.11.019 30581017 PMC6335594

[B54] Nelson S. B. Valakh V. (2015). Excitatory/Inhibitory balance and circuit homeostasis in autism spectrum disorders. *Neuron* 87 684–698. 10.1016/j.neuron.2015.07.033 26291155 PMC4567857

[B55] Northrup H. Krueger D. A. (2013). Tuberous sclerosis complex diagnostic criteria update: recommendations of the 2012 iinternational tuberous sclerosis complex consensus conference. *Pediatr. Neurol.* 49 243–254. 10.1016/j.pediatrneurol.2013.08.001 24053982 PMC4080684

[B56] Ohnishi T. Matsuda H. Hashimoto T. Kunihiro T. Nishikawa M. Uema T. (2000). Abnormal regional cerebral blood flow in childhood autism. *Brain* 123(Pt 9), 1838–1844. 10.1093/brain/123.9.1838 10960047

[B57] Overwater I. E. Rietman A. B. Mous S. E. Bindels-de Heus K. Rizopoulos D. Ten Hoopen L. W. (2019). A randomized controlled trial with everolimus for IQ and autism in tuberous sclerosis complex. *Neurology* 93 e200–e209. 10.1212/WNL.0000000000007749 31217257

[B58] Palva S. Palva J. M. (2007). New vistas for alpha-frequency band oscillations. *Trends Neurosci.* 30 150–158. 10.1016/j.tins.2007.02.001 17307258

[B59] Peng C. K. Havlin S. Stanley H. E. Goldberger A. L. (1995). Quantification of scaling exponents and crossover phenomena in nonstationary heartbeat time series. *Chaos* 5 82–87. 10.1063/1.166141 11538314

[B60] Poil S. S. Hardstone R. Mansvelder H. D. Linkenkaer-Hansen K. (2020). Critical-state dynamics of avalanches and oscillations jointly emerge from balanced excitation/inhibition in neuronal networks. *J. Neurosci.* 32 9817–9823. 10.1523/JNEUROSCI.5990-11.2012 22815496 PMC3553543

[B61] Prather P. de Vries P. J. (2004). Behavioral and cognitive aspects of tuberous sclerosis complex. *J. Child Neurol.* 19 666–674. 10.1177/08830738040190090601 15563012

[B62] Puskarjov M. Kahle K. T. Ruusuvuori E. Kaila K. (2014). Pharmacotherapeutic targeting of cation-chloride cotransporters in neonatal seizures. *Epilepsia* 55 806–818. 10.1111/epi.12620 24802699 PMC4284054

[B63] Rajpurkar P. Yang J. Dass N. Vale V. Keller A. S. Irvin J. (2020). Evaluation of a machine learning model based on pretreatment symptoms and electroencephalographic features to predict outcomes of antidepressant treatment in adults with depression: a prespecified secondary analysis of a randomized clinical trial. *JAMA Netw Open* 3:e206653. 10.1001/jamanetworkopen.2020.6653 32568399 PMC7309440

[B64] Ricci L. Assenza G. Pulitano P. Simonelli V. Vollero L. Lanzone J. (2021). Measuring the effects of first antiepileptic medication in temporal lobe epilepsy: predictive value of quantitative-EEG analysis. *Clin. Neurophysiol.* 132 25–35. 10.1016/j.clinph.2020.10.020 33248432

[B65] Ruffolo G. Iyer A. Cifelli P. Roseti C. Mühlebner A. van Scheppingen J. (2016). Functional aspects of early brain development are preserved in tuberous sclerosis complex (TSC) epileptogenic lesions. *Neurobiol. Dis.* 95 93–101. 10.1016/j.nbd.2016.07.014 27425893

[B66] Sahin M. (2012). Targeted treatment trials for tuberous sclerosis and autism: no longer a dream. *Curr. Opin. Neurobiol.* 22 895–901. 10.1016/j.conb.2012.04.008 22560338 PMC3715752

[B67] Smith R. J. Sugijoto A. Rismanchi N. Hussain S. A. Shrey D. W. Lopour B. A. (2017). Long-Range temporal correlations reflect treatment response in the electroencephalogram of patients with infantile spasms. *Brain Topogr.* 30 810–821. 10.1007/s10548-017-0588-5 28905146 PMC6058722

[B68] Sprengers J. J. van Andel D. M. Zuithoff N. P. A. Keijzer-Veen M. G. Schulp A. J. A. Scheepers F. E. (2020). Bumetanide for core symptoms of autism spectrum disorder (BAMBI): a single center, double-blinded, participant-randomized, placebo-controlled, Phase-2 superiority trial. *J. Am. Acad. Child Adolesc. Psychiatry* 60 865–876. 10.1016/j.jaac.2020.07.888 32730977

[B69] Stoner R. Chow M. L. Boyle M. P. Sunkin S. M. Mouton P. R. Roy S. (2014). Patches of disorganization in the neocortex of children with autism. *N. Engl. J. Med.* 370 1209–1219. 10.1056/NEJMoa1307491 24670167 PMC4499461

[B70] Taki M. M. Harada M. Mori K. Kubo H. Nose A. Matsuda T. (2009). High gamma-aminobutyric acid level in cortical tubers in epileptic infants with tuberous sclerosis complex measured with the MEGA-editing J-difference method and a three-Tesla clinical MRI Instrument. *Neuroimage* 47 1207–1214. 10.1016/j.neuroimage.2009.05.060 19481612

[B71] Talos D. M. Sun H. Kosaras B. Joseph A. Folkerth R. D. Poduri A. (2012). Altered inhibition in tuberous sclerosis and type IIb cortical dysplasia. *Ann. Neurol.* 71 539–551. 10.1002/ana.22696 22447678 PMC3334406

[B72] Tao J. X. Chen X. J. Baldwin M. Yung I. Rose S. Frim D. (2011). Interictal regional delta slowing is an EEG marker of epileptic network in temporal lobe epilepsy. *Epilepsia* 52 467–476. 10.1111/j.1528-1167.2010.02918.x 21204828

[B73] Terney D. Alving J. Skaarup C. N. Wolf P. Beniczky S. (2010). The slow-wave component of the interictal epileptiform EEG discharges. *Epilepsy Res.* 90 228–233. 10.1016/j.eplepsyres.2010.05.005 20554157

[B74] Tierney A. L. Gabard-Durnam L. Vogel-Farley V. Tager-Flusberg H. Nelson C. A. (2012). Developmental trajectories of resting EEG power: an endophenotype of autism spectrum disorder. *PLoS One* 7:e39127. 10.1371/journal.pone.0039127 22745707 PMC3380047

[B75] Turrigiano G. G. Nelson S. B. (2004). Homeostatic plasticity in the developing nervous system. *Nat. Rev. Neurosci.* 5 97–107. 10.1038/nrn1327 14735113

[B76] Tyzio R. Nardou R. Ferrari D. C. Tsintsadze T. Shahrokhi A. Eftekhari S. (2014). Oxytocin-mediated GABA inhibition during delivery attenuates autism pathogenesis in rodent offspring. *Science* 343 675–679. 10.1126/science.1247190 24503856

[B77] van Andel D. M. Sprengers J. J. Oranje B. Scheepers F. E. Jansen F. E. Bruining H. (2020). Effects of bumetanide on neurodevelopmental impairments in patients with tuberous sclerosis complex: an open-label pilot study. *Mol. Autism.* 11:30. 10.1186/s13229-020-00335-4 32381101 PMC7204231

[B78] Vlaskamp C. Poil S. S. Jansen F. Linkenkaer-Hansen K. Durston S. Oranje B. (2017). Bumetanide as a candidate treatment for behavioral problems in tuberous sclerosis complex. *Front. Neurol.* 8:469. 10.3389/fneur.2017.00469 28943860 PMC5596068

[B79] Wang J. Barstein J. Ethridge L. E. Mosconi M. W. Takarae Y. Sweeney J. A. (2013). Resting state EEG abnormalities in autism spectrum disorders. *J. Neurodev. Disord.* 5:24. 10.1186/1866-1955-5-24 24040879 PMC3847481

[B80] Wang Y. Greenwood J. S. Calcagnotto M. E. Kirsch H. E. Barbaro N. M. Baraban S. C. (2007). Neocortical hyperexcitability in a human case of tuberous sclerosis complex and mice lacking neuronal expression of TSC1. *Ann. Neurol.* 61 139–152. 10.1002/ana.21058 17279540

[B81] White R. Hua Y. Scheithauer B. Lynch D. R. Henske E. P. Crino P. B. (2001). Selective alterations in glutamate and GABA receptor subunit mRNA expression in dysplastic neurons and giant cells of cortical tubers. *Ann. Neurol.* 49 67–78. 10.1002/1531-8249(200101)49:1<67::aid-ana10>3.0.co;2-l 11198298

[B82] Wu W. Zhang Y. Jiang J. Lucas M. V. Fonzo G. A. Rolle C. E. (2020). An electroencephalographic signature predicts antidepressant response in major depression. *Nat. Biotechnol.* 38 439–447. 10.1038/s41587-019-0397-3 32042166 PMC7145761

[B83] Zhang L. Huang C. C. Dai Y. Luo Q. Ji Y. Wang K. (2020). Symptom improvement in children with autism spectrum disorder following bumetanide administration is associated with decreased GABA/glutamate ratios. *Transl. Psychiatry* 10:9. 32066666 10.1038/s41398-020-0692-2 PMC7026137

[B84] Zhdanov A. Atluri S. Wong W. Vaghei Y. Daskalakis Z. J. Blumberger D. M. (2020). Use of machine learning for predicting escitalopram treatment outcome from electroencephalography recordings in adult patients with depression. *JAMA Netw. Open* 3:e1918377. 10.1001/jamanetworkopen.2019.18377 31899530 PMC6991244

