# Supplementary material for: Bumetanide Effects on Resting-State EEG in Tuberous Sclerosis Complex in Relation to Clinical Outcome: An Open-Label Study
Source: Front Neurosci. 2022 May 12;16:879451. doi: 10.3389/fnins.2022.879451 (PMC9134117; doi:10.3389/fnins.2022.879451)
Supplement: Supplementary file 1 [file Data_Sheet_1.docx]

**Supplementary Material**

**Bumetanide effects on resting-state EEG in tuberous sclerosis complex in relation to clinical outcome: an open-label study**

1. **Supplementary Figure 1.** Participants with TSC show lower regional absolute power and *f*E/I than TDC.
2. **Supplementary Table 1.** Subjects included for EEG analysis, demographics and clinical scores.
3. **Supplementary Table 2.** Subjects included for EEG analysis and their EEG measures

**Supplementary Figures**


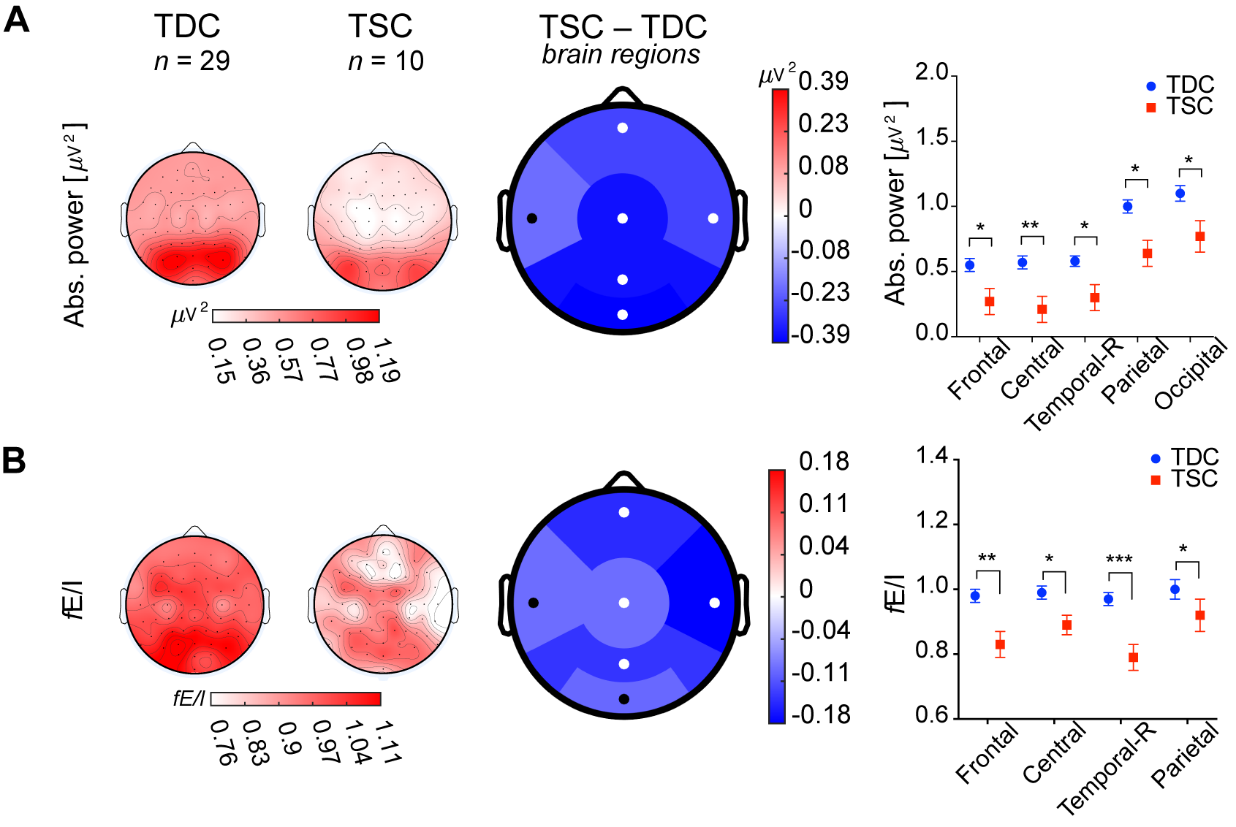


**Supplementary Figure 1. Participants with TSC show lower regional absolute power and *f*E/I than TDC. A)** The TSC group had lower absolute power than the TDC in five out of the six brain regions (frontal, central, temporal right, parietal and occipital), particularly in the central region. **B)** *f*E/I was also lower in TSC when compared to TDC in four out of six brain regions, particularly in frontal and temporal right regions. As in Figure 2, grand-average topographies for the EEG biomarkers are shown for TDC (first column), TSC (D0; second column), and comparisons by brain regions –TSC-minus-TDC (third column; frontal, temporal left, temporal right, central, parietal and occipital). White circles represent significant regions (*p*-value < 0.05, Wilcoxon rank-sum test, FDR corrected). Comparisons represented in graph (fourth column) are based on the average value of the EEG biomarkers across the channels contained in each region (mean ± SEM; see Methods). (*) *p* < .05, (**) *p* < .01, (***) *p* < .001.

**Supplementary Tables**

|  | ID | AGE | Sex | MED | ABC-I  D0 | ABC-I  D91 | ABC-I  D119 | SRS  D0 | SRS  D91 | SRS  D119 | RBS  D0 | RBS  D91 | RBS  D119 |
| --- | --- | --- | --- | --- | --- | --- | --- | --- | --- | --- | --- | --- | --- |
| TSC |  |  |  |  |  |  |  |  |  |  |  |  |  |
| 1 | T001† | 16.3 | fem | other | 6 | 6 | 1 | 93 | 60 | 60 | 7 | 2 | 1 |
| 2 | T002†* | 13.8 | fem | AED | 17 | 5 | 8 | 119 | 84 | 70 | 17 | 4 | 9 |
| 3 | T007†* | 11.2 | male | AED | 24 | 13 | 11 | 108 | 69 | 84 | 55 | 43 | 50 |
| 4 | T008† | 17.6 | fem | other | 14 | 0 | 5 | 37 | 36 | 26 | 8 | 3 | 8 |
| 5 | T009†* | 21.3 | male | AED | 21 | 17 | 14 | 78 | 52 | 66 | 9 | 3 | 5 |
| 6 | T010†* | 10.7 | male | AED | 3 | 2 | 3 | 42 | 39 | 50 | 3 | 3 | 9 |
| 7 | T011† | 9.5 | male | none | 10 | 4 | 7 | 80 | 52 | 61 | 16 | 6 | 9 |
| 8 | T012†* | 8.3 | male | AED | 12 | 2 | 2 | 47 | 43 | 48 | 0 | 2 | 0 |
| 9 | T015†* | 13.7 | fem | none | 4 | 1 | 2 | 68 | 59 | 56 | 11 | 10 | 11 |
| 10 | T017† | 11.8 | fem | none | 11 | 4 | 10 | 72 | 67 | 74 | 12 | 15 | 16 |
| TDC |  |  |  |  |  |  |  |  |  |  |  |  |  |
| 1 | SP011 | 9.6 | male | none | 1 |  |  | 17 |  |  | 2 |  |  |
| 2 | SP029 | 10.0 | fem | none | 1 |  |  | 11 |  |  | 0 |  |  |
| 3 | SP046 | 12.0 | fem | none | 4 |  |  | 23 |  |  | 4 |  |  |
| 4 | SP062 | 12.1 | male | none | 2 |  |  | 8 |  |  | 0 |  |  |
| 5 | SP077 | 10.5 | fem | none | 1 |  |  | 25 |  |  | 0 |  |  |
| 6 | SP079 | 11.5 | fem | none | 0 |  |  | 18 |  |  | 1 |  |  |
| 7 | SP080 | 12.4 | fem | none | 0 |  |  | 17 |  |  | 0 |  |  |
| 8 | SP094 | 10.6 | fem | none | 0 |  |  | 5 |  |  | 0 |  |  |
| 9 | SP095 | 9.3 | fem | none | 0 |  |  | 5 |  |  | 0 |  |  |
| 10 | SP096 | 9.3 | fem | none | 0 |  |  | 10 |  |  | 0 |  |  |
| 11 | SP097 | 11.8 | fem | none | 0 |  |  | 12 |  |  | 0 |  |  |
| 12 | SP100 | 10.1 | male | none | 1 |  |  | 26 |  |  | 3 |  |  |
| 13 | SP101 | 10.0 | male | none | 0 |  |  | 13 |  |  | 0 |  |  |
| 14 | SP108 | 9.6 | fem | none | 2 |  |  | 18 |  |  | 0 |  |  |
| 15 | SP110 | 10.6 | fem | none | 1 |  |  | 18 |  |  | 0 |  |  |
| 16 | SP111 | 10.3 | fem | none | 0 |  |  | 30 |  |  | 0 |  |  |
| 17 | SP117 | 9.6 | male | none |  |  |  |  |  |  |  |  |  |
| 18 | SP118 | 7.8 | fem | none | 6 |  |  | 15 |  |  | 0 |  |  |
| 19 | SP120 | 11.0 | male | none | 0 |  |  | 10 |  |  | 0 |  |  |
| 20 | SP129 | 11.2 | male | none | 1 |  |  | 11 |  |  | 0 |  |  |
| 21 | SP131 | 10.8 | male | none | 1 |  |  | 18 |  |  | 0 |  |  |
| 22 | SP136 | 11.2 | male | none | 0 |  |  | 13 |  |  | 1 |  |  |
| 23 | SP137 | 7.4 | fem | none | 0 |  |  | 23 |  |  | 3 |  |  |
| 24 | SP139 | 7.6 | male | none | 0 |  |  | 17 |  |  | 0 |  |  |
| 25 | SP140 | 10.5 | male | none | 0 |  |  | 30 |  |  | 6 |  |  |
| 26 | SP142 | 8.9 | male | none | 5 |  |  | 39 |  |  | 0 |  |  |
| 27 | SP143 | 8.2 | male | none | 0 |  |  | 22 |  |  | 3 |  |  |
| 28 | SP144 | 10.2 | male | none | 2 |  |  | 14 |  |  | 1 |  |  |
| 29 | SP147 | 14.5 | fem | none | 0 |  |  | 28 |  |  | 0 |  |  |

**Supplementary Table 1. Subjects included for EEG analysis, demographics and clinical scores**. TSC (Tuberous Sclerosis Complex). TDC (Typically Developing Children). ABC-I (Aberrant Behavior Checklist Irritability subscale). SRS (social responsiveness scale- 2). RBS (Repetitive Behavioral Scale-Revised). D0 -day-zero baseline recording, D91 -day 91 of treatment, D119 -day 119 (after 28-days washout period). † Cortical tubers. *Epilepsy comorbidity. MED (Medication). AED (Antiepileptic drug). Blanks (non-applicable (TDC)).

|  | ID | AP  D0 | AP  D91 | AP  D119 | DFA  D0 | DFA  D91 | DFA  D119 | *f*E/I  D0 | *f*E/I  D91 | *f*E/I  D119 |
| --- | --- | --- | --- | --- | --- | --- | --- | --- | --- | --- |
| TSC |  |  |  |  |  |  |  |  |  |  |
| 1 | T001 | 0.99 | 1.48 | 1.46 | 0.62 | 0.74 | 0.74 | 0.77 | 0.79 | 0.78 |
| 2 | T002 | 3.73 | 5.88 | 3.38 | 0.75 | 0.73 | 0.76 | 0.86 | 0.95 | 0.88 |
| 3 | T007 | 3.44 | 3.01 | 3.01 | 0.71 | 0.80 | 0.78 | 0.99 | 0.88 | 1.01 |
| 4 | T008 | 1.30 | 2.29 | 1.01 | 0.72 | 0.71 | 0.79 | 0.83 | 1.05 | 0.89 |
| 5 | T009 | 3.01 | M | M | 0.57 | M | M | 0.96 | M | M |
| 6 | T010 | 1.70 | 2.04 | 1.28 | 0.67 | 0.79 | 0.79 | 0.76 | 0.83 | 0.69 |
| 7 | T011 | 8.18 | 9.76 | 6.10 | 0.61 | 0.61 | 0.68 | 1.07 | 1.05 | 1.07 |
| 8 | T012 | 8.38 | 7.98 | 9.52 | 0.70 | 0.80 | 0.76 | 0.90 | 0.83 | 0.89 |
| 9 | T015 | 1.20 | 1.14 | 1.12 | 0.67 | 0.67 | 0.68 | 0.82 | 0.74 | 0.81 |
| 10 | T017 | 4.72 | 4.75 | 4.22 | 0.61 | 0.64 | 0.64 | 0.83 | 0.89 | 0.92 |
| TDC |  |  |  |  |  |  |  |  |  |  |
| 1 | SP011 | 5.01 |  |  | 0.69 |  |  | 1.05 |  |  |
| 2 | SP029 | 3.99 |  |  | 0.66 |  |  | 0.96 |  |  |
| 3 | SP046 | 3.82 |  |  | 0.67 |  |  | 0.88 |  |  |
| 4 | SP062 | 25.23 |  |  | 0.66 |  |  | 1.22 |  |  |
| 5 | SP077 | 3.26 |  |  | 0.64 |  |  | 0.97 |  |  |
| 6 | SP079 | 13.62 |  |  | 0.66 |  |  | 1.08 |  |  |
| 7 | SP080 | 2.61 |  |  | 0.62 |  |  | 0.76 |  |  |
| 8 | SP094 | 8.63 |  |  | 0.67 |  |  | 1.12 |  |  |
| 9 | SP095 | 5.54 |  |  | 0.62 |  |  | 0.99 |  |  |
| 10 | SP096 | 5.51 |  |  | 0.70 |  |  | 1.04 |  |  |
| 11 | SP097 | 9.91 |  |  | 0.66 |  |  | 1.02 |  |  |
| 12 | SP100 | 11.94 |  |  | 0.74 |  |  | 1.05 |  |  |
| 13 | SP101 | 6.88 |  |  | 0.60 |  |  | 1.06 |  |  |
| 14 | SP108 | 10.27 |  |  | 0.70 |  |  | 1.01 |  |  |
| 15 | SP110 | 8.43 |  |  | 0.61 |  |  | 0.95 |  |  |
| 16 | SP111 | 4.47 |  |  | 0.66 |  |  | 0.87 |  |  |
| 17 | SP117 | 5.01 |  |  | 0.63 |  |  | 1.10 |  |  |
| 18 | SP118 | 2.21 |  |  | 0.65 |  |  | 0.92 |  |  |
| 19 | SP120 | 14.34 |  |  | 0.56 |  |  | 1.04 |  |  |
| 20 | SP129 | 7.62 |  |  | 0.60 |  |  | 1.27 |  |  |
| 21 | SP131 | 3.57 |  |  | 0.72 |  |  | 0.92 |  |  |
| 22 | SP136 | 5.99 |  |  | 0.67 |  |  | 0.97 |  |  |
| 23 | SP137 | 4.44 |  |  | 0.68 |  |  | 0.92 |  |  |
| 24 | SP139 | 16.03 |  |  | 0.70 |  |  | 1.14 |  |  |
| 25 | SP140 | 5.89 |  |  | 0.68 |  |  | 1.17 |  |  |
| 26 | SP142 | 3.86 |  |  | 0.63 |  |  | 1.04 |  |  |
| 27 | SP143 | 9.08 |  |  | 0.69 |  |  | 0.96 |  |  |
| 28 | SP144 | 3.02 |  |  | 0.62 |  |  | 0.92 |  |  |
| 29 | SP147 | 2.83 |  |  | 0.67 |  |  | 0.93 |  |  |

**Supplementary Table 2. Subjects included for EEG analysis and their EEG measures**. TSC (Tuberous Sclerosis Complex). TDC (Typically Developing Children). AP (absolute power). DFA (scaling exponent of the detrended fluctuation analysis). *fE*/*I* (excitation-inhibition ratio). M (missing EEG measure –EEG not available). D0 -day-zero baseline recording, D91 -day 91 of treatment, D119 -day 119 (after 28-days washout period). Blanks (non-applicable (TDC)).
